# Supplementary material for: Measurement of breast artery calcification using an artificial intelligence detection model and its association with major adverse cardiovascular events
Source: PLOS Digit Health. 2024 Dec 23;3(12):e0000698. doi: 10.1371/journal.pdig.0000698 (PMC11665981; doi:10.1371/journal.pdig.0000698)
Supplement: S1 Table — ASCVD = Atherosclerotic Cardiovascular Disease, BAC = Breast artery calcification, CAC = coronary artery calcification. *Fisher’s exact test. (DOCX) [file pdig.0000698.s001.docx]

| Variable | Category | ASCVD risk score | | Chi Square  P-value |
| --- | --- | --- | --- | --- |
|  |  | Low risk (<7.5%)  n=29 | Moderate - High risk (7.5% - over 20%)  n=59 |  |
| CAC | *No* | 24 (82.76%) | 32 (54.24%) | 0.009 |
|  | *Yes* | 5(17.24%) | 27 (45.76%) |  |
| BAC | *No* | 22 (75.86%) | 25 (42.37%) | 0.003 |
|  | *Yes* | 7(24.14%) | 34 (57.63%) |  |

**S1 Table**: Association of BAC, CAC with ASCVD risk score (n=99). ASCVD = Atherosclerotic Cardiovascular Disease, BAC = Breast artery calcification, CAC = coronary artery calcification. *Fisher's exact test
