# Supplementary material for: Measurement of breast artery calcification using an artificial intelligence detection model and its association with major adverse cardiovascular events
Source: PLOS Digit Health. 2024 Dec 23;3(12):e0000698. doi: 10.1371/journal.pdig.0000698 (PMC11665981; doi:10.1371/journal.pdig.0000698)
Supplement: S2 Table — ASCVD = Atherosclerotic Cardiovascular Disease, BAC = Breast artery calcification, CAC = coronary artery calcification, CI = Confidence Interval, OR = Odds Ratio. (DOCX) [file pdig.0000698.s002.docx]

| Variable | OR (95% CI) |
| --- | --- |
| CAC (yes vs no) | 4.05 (1.36, 12.06) |
| BAC (yes vs no) | 4.27 (1.58-11.56) |
| CAC (continuous) | 1.19 (1.0, 1.44) |
| BAC (continuous) | 1.06 (1.01, 1.15) |

**S2 Table**: Odds Ratios of moderate-high ASCVD risk score (n=99). ASCVD = Atherosclerotic Cardiovascular Disease, BAC = Breast artery calcification, CAC = coronary artery calcification, CI = Confidence Interval, OR = Odds Ratio.
