# Supplementary material for: Measurement of breast artery calcification using an artificial intelligence detection model and its association with major adverse cardiovascular events
Source: PLOS Digit Health. 2024 Dec 23;3(12):e0000698. doi: 10.1371/journal.pdig.0000698 (PMC11665981; doi:10.1371/journal.pdig.0000698)
Supplement: S3 Table — ASCVD = Atherosclerotic Cardiovascular Disease, BAC = Breast artery calcification, CAC = coronary artery calcification, SD = Standard Deviation. (DOCX) [file pdig.0000698.s003.docx]

| Variable | ASCVD Mean (SD) | | T-test P-value |
| --- | --- | --- | --- |
|  | Low risk (<7.5%)  n=29 | Moderate - High risk (7.5% - over 20%)  n=59 |  |
| CAC | 1.31 (2.87) | 2.83 (3.42) | 0.04 |
| BAC | 3.78 (8.54) | 27.38 (33.62) | <0.0001 |

**S3 Table**: Mean Score among ASCVD risk score (yes/no) (n=99). ASCVD = Atherosclerotic Cardiovascular Disease, BAC = Breast artery calcification, CAC = coronary artery calcification, SD = Standard Deviation.
