# Supplementary material for: Measurement of breast artery calcification using an artificial intelligence detection model and its association with major adverse cardiovascular events
Source: PLOS Digit Health. 2024 Dec 23;3(12):e0000698. doi: 10.1371/journal.pdig.0000698 (PMC11665981; doi:10.1371/journal.pdig.0000698)
Supplement: Attachment — Submitted filename: Response to Reviewers.docx [file pdig.0000698.s004.docx]

| Comment | Response |
| --- | --- |
| Reviewer #1 | |
| I recommend restructuring the study to compare the diagnostic utility of ASCVD risk score vs BAC vs CAC in MACE prediction. Associations between ASCVD risk and BAC/CAC are not of clinical utility in my opinion. | Thank you for your comments. Our results provide evidence to support the potential utilization of AI generated BAC measurements from standard of care mammograms in addition to the widely adopted ASCVD and CAC scores, to identify and risk-stratify women who are at increased risk of CVD and may benefit from targeted prevention measures. BAC at the current time remains an investigational tool, which can potentially augment CV risk prediction in women patients, in addition to the guideline suported CAC and ASCVD risk score. |
| Also, a key limitation here is that non-gated CT chest was used to classify CAC, not a gated cardiac CT. | Thank you this has been included as a limitation with a statement added regarding our previous published manuscript which attested to the clinical utility of using non-gated CT )Weston method developed by Cleveland Clinic to measure CAC^1^. As most patients need to pay out of pocket for the gated cardiac CT, the method we previously described allows for scoring of CAC from a non-gated chest CT, which does not cost the patient any additional out-of-pocket expenses. We will adopt a new AI based algorhym for CAC quantitation in the future |
| ***Reviewer #2:*** | |
| 1. About the AI model in the method section: more details should be included about this model. For example, the authors claimed that over 20,000 mammograms were used in the training. What percentage of those images has BAC? How was the true BAC score determined for the training data set. Is there any validation at all for the test data set used in this study? | Thank you for the request for additional clarification. We have provided a detailed description of the AI model in the methods. We welcome the reviewers feedback of this updated section. |
| 2. The authors did not clarify it, but it seems to suggest that both 2D and TOMO images were used for BAC assessment in this study. Were there any differences in the findings, in regard to the use of 2D or TOMO images? | To clarify, in the present study of approximately 100 patients, we only used 2D images from mammography standard of care tests to score the breast arterial calcification. The non-gated chest CT images were also gathered from standard of care imaging and a calcium score assessed as previously reported^1^. The present study includes patients from a single site. |
| 3. The authors used a Bradley Score of 5 to classify the BAC images into a binary category. But there was no discussion about how this threshold was chosen? Is the associations between BAC and MACE sensitive to this threshold setting? | Thank you for the request for additional clarification. We have provided a detailed description of the AI model in the methods. We welcome the reviewers feedback of this updated section.  In addition, in the spirit of scientific pursuit, we did re-analyze the data with a threshold of 10, which did not change the results significantly. We have included the tables here with that threshold limit. We welcome the reviewer’s feedback if they would also like this data to be included in the paper. For most of our analysis we have used Bradley (continuous variable), so using >10 will have no effect on the results with MACE. The odds ratio for BAC>10 was very high than BAC>5. Please see Tables 1 and 2 and Supplemental Tables 1 and 2. |
| 4. The authors suggested that each unit increase in Bradley score in BAC has a 6% increase of odds in ASCVD. How about the increased risk of MACE? | Please see table 5 in the revised manuscript which has been added in to display the odds ratios ascertained. |
| 5. In page 14, the authors claimed that the combination of positive BAC and positive ASCVD correlates strongly with MACE. However, the reviewer was not able to find direct results to support this conclusion. | Upon careful review, we understand the reviewer’s concerns and agree that the data does not support this statement. We have revised the statement. |
| **Editor Comments:** | |
| We do not publish any copyright or trademark symbols that usually accompany proprietary names, eg (R), (C), or TM (e.g. next to drug or reagent names). Please remove all instances of trademark/copyright symbols throughout the text, including ® and ™ on pages 17 and 7. | Thank you – these have been removed throughout. |
| We noticed that you used "data not shown" in the manuscript. We do not allow these references, as the PLOS data access policy requires that all data be either published with the manuscript or made available in a publicly accessible database. Please amend the supplementary material to include the referenced data or remove the references. | This data has been added into the manuscript as Table 4. |
| In the online submission form, you indicated that your data will be submitted to a repository upon acceptance. We strongly recommend all authors deposit their data before acceptance, as the process can be lengthy and hold up publication timelines. Please note that, though access restrictions are acceptable now, your entire minimal dataset will need to be made freely accessible if your manuscript is accepted for publication. This policy applies to all data except where public deposition would breach compliance with the protocol approved by your research ethics board. If you are unable to adhere to our open data policy, please kindly revise your statement to explain your reasoning and we will seek the editor's input on an exemption. | We have made the data publicly available:  <https://doi.org/10.6084/m9.figshare.26852719.v1>.^2^  A statement to this nature has also been added to the manuscript under data collection.  Please note that when the data was reviewed again in entirety, we realized a discrepancy between the total number of patients reported in the first version of the manuscript with BAC. This has been updated throughout and did not have an impact on any statistical significance. |
| To the reviewers' comments, I would also add the request to enhance the manuscript in order to highlight better the significance of the study. | The authors have made significant changes to the manuscript. We welcome your feedback to these changes. |
| Was the study protocol and statistical analysis plan registered before the start of the study? | The protocol and statistical analysis were not registered before the start of the study. Standard procedure at our community based-teaching hospital when performing a retrospective chart review is to obtain IRB approval prior to study initiation but not to register as this was not an  interventional study or randomized clinical trial. The current study was granted exemption from the IRB. |
| Having tables split over two pages make it difficult to read. Please make sure this does not happen in any resubmission. | We have corrected in this version. Thank you. |
| cover page: I do understand the word "objective" in the sentence "Objective Breast artery calcification (BAC) obtained from standard mammographic  images is currently under evaluation to stratify risk of major cardiovascular events in  women." | Thank you – in reviewing the document as it was created, there were formatting issues which have been addressed in this current submission. |
| Abstract p.2 spell out ASCVD and include this variable in the objctive section.  "Contingency tables, Chi-square tests and/or Fisher's exact  tests assessed the associations between arterial calcifications and categorical variables of interest" the result of those test are not reported nor commented in the main text. | Thank you – we have updated the abstract. |
| Key messages p.4: "It has previously been reported an association exists between breast artery calcification and coronary artery calcification, the latter of which is associated with coronary artery disease".  In what way does a message reported in earlier studies constitute a key message of the present study? | We have updated the key message to be more specific to the present study. |
| "Using artificial intelligence to measure breast artery calcification"  If artificial intelligence is used, I would call it an "estimate" rather than a "measurement". | This has been updated – thank you. |
| p.6 Study Design and Methods  - What is the follow up period for the observation of MACE?  - How was censoring handled? | Please see sentence added under Study Population to clarify the follow-up period for the observation of MACE.  This is not a prospective randomized controlled trial – it was retrospective in review so we included all patients that met inclusion criteria and all were followed for MACE.  It is not the type of analysis we established in the protocol. We have added a sentence under study population as clarification. |
| p.7 Description of the Artificial Intelligence (AI) Model  Is the ML approach explained in more detail elsewhere? | We have provided a detailed description of the AI model in the methods. In addition, two references have been added describing a small pilot study (references from recent conference proceedings). ^3,4^ |
| p. 8-9 "Contingency tables, chi-square tests and/or Fisher's exact tests were  performed to assess the association between breast and coronary arterial calcifications and categorical variables of interest (BAC vs MACE, CAC vs MACE, ASCVD risk vs MACE)."  There are four variables involved here, hence up to six pairwise comparisons.  What was the rationale for carrying out only those three comparisons? | We have added clarification and updates to the manuscript. |
| p.9 "Risk differences were calculated to capture the difference in observed risk between the reference groups and the groups with the exposure of interest."  At this stage of my perusal, it was not clear "the exposure of interest" referred to. | We have added clarification and updates to the manuscript. |
| p. 10-11 It is not clear what Table 2 contains.  I have the impression that the right most column contain risk values and risk difference values. Please enhance wording in cells and caption.  Caption mentions "ASCVD" in the sentence "Risk difference of MACE on BAC, CAC and ASCVD" but I have the impression that throughout the paper, it is referred to "ASCVD risk". | We have added clarification and updates to the manuscript. |
| p. 11-12 as pointed out earlier, the description of the logistic regression modelling lacks details | We have added clarification and updates to the manuscript. |

References:

1.	Chen L, Vavrenyuk A, Ren JH, et al. Prognostic Value of Coronary Artery Calcification Identified by the Semi-quantitative Weston Method in the Emergency Room or Other Hospitalized Patients. *Front Cardiovasc Med*. 2021;8. doi:10.3389/fcvm.2021.684292

2.	Suzanne Rose. BAC Paper_Deidentified_Figshare.xlsx. Published online August 28, 2024. doi:10.6084/m9.figshare.26852719.v1

3.	AI flags breast arterial calcification on mammography. AuntMinnie. May 22, 2022. Accessed August 27, 2024. https://www.auntminnie.com/clinical-news/womens-imaging/breast/article/15631053/ai-flags-breast-arterial-calcification-on-mammography

4.	Browse by Speaker. Accessed August 27, 2024. https://www.eventscribe.net/2022/SBIACR2022/speakers.asp?pfp=BrowsebySpeaker

| Variable | Category | Count (%) |
| --- | --- | --- |
| Age | *40-49* | 6 (6.06%) |
|  | *50-59* | 16 (16.16%) |
|  | *60-69* | 30 (30.30%) |
|  | *70 and over* | 47 (47.47%) |
| Race | *White* | 68 (68.69%) |
|  | *Black/ African American* | 16 (16.16%) |
|  | *Hispanic/Latino* | 12 (12.12%) |
|  | *Other* | 3 (3.03%) |
| BAC >5 | *Positive* | 49 (49.49%) |
|  | *Negative* | 50 (50.51%) |
| BAC >10 | *Positive*  *Negative* | 39 (39.39%)  60 (60.61%) |
| CAC | *Positive* | 37 (37.37%) |
|  | *Negative* | 62 (62.63%) |
| ASCVD* | *Low risk (<5%)* | 22 (25.0%) |
|  | *Borderline risk (5%-7.4%)* | 7 (7.95%) |
|  | *Intermediate risk (7.5%-19.9%)* | 30 (34.09%) |
|  | *High risk (>20%)* | 29 (32.95%) |
| ASCVD* (binary) | *Low risk (<7.5%)* | 29 (32.95%) |
|  | *Moderate – High risk (7.5% - over 20%)* | 59 (67.05%) |
| MACE | *Yes* | 26 (26.26%) |
|  | *No* | 73 (73.74%) |
| Death | *Yes* | 16 (16.16%) |
|  | *No* | 83 (83.84%) |
| Smoking Status | *Never* | 57 (57.58%) |
|  | *Current* | 38 (38.38%) |
|  | *Unknown* | 4 (4.04%) |
| Diabetes | *Yes* | 20 (20.20%) |
|  | *No* | 79 (79.80%) |
| Hypertension | *Yes* | 58 (58.58%) |
|  | *No* | 41 (41.41%) |

**Table 1:** Overall Demographics and clinical characteristics (n=99). ASCVD = Atherosclerotic Cardiovascular Disease, BAC = Breast artery calcification, CAC = coronary artery calcification, MACE = Major Adverse Cardiac Events. *ASCVD risk score was incalculable for 11 patients due to data outside the allowable thresholds.

| Variable | Category | Risk value (95% CI) |
| --- | --- | --- |
| CAC | *No* | 0.21 (0.09, 0.33) |
|  | *Yes* | 0.30 (0.18,0.42) |
| **CAC Absolute Risk Difference** | | 0.09 (-0.08, 0.26) |
| BAC | *No* | 0.18 (0.7-0.28) |
|  | *Yes* | 0.35 (0.21-0.48) |
| **BAC Absolute Risk Difference** | | 0.17 (0.08-0.33) |
| **BAC>10 risk diff** | | 0.28 (0.09-0.10) |
| ASCVD risk score | Low risk (<7.5%) | 0.03 (0.0,0.10) |
|  | Moderate – High risk (7.5% - over 20%) | 0.32 (0.20,0.44) |
| **ASCVD risk score Absolute Risk Difference** | | 0.28 (0.15,0.42) |

**Table 2:** Risk value and risk difference of MACE on BAC, CAC and ASCVD risk (n=99). ASCVD = Atherosclerotic Cardiovascular Disease, BAC = Breast artery calcification, CAC = coronary artery calcification, CI = Confidence Interval.

**Supplemental Material**

| Variable | Category | ASCVD risk score | | Chi Square  P-value |
| --- | --- | --- | --- | --- |
|  |  | Low risk (<7.5%)  n=29 | Moderate - High risk (7.5% - over 20%)  n=59 |  |
| CAC | *No* | 24 (82.76%) | 32 (54.24%) | 0.009 |
|  | *Yes* | 5(17.24%) | 27 (45.76%) |  |
| BAC | *No* | 22 (75.86%) | 25 (42.37%) | 0.003 |
|  | *Yes* | 7(24.14%) | 34 (57.63%) |  |
| BAC >10 | *no*  *yes* | 27 (93.10%)  2 (6.90%) | 30 (50.85%)  29 (49.15%) | <0.0001* |

**Supplemental Table 1**: Association of BAC, CAC with ASCVD risk score (n=99). ASCVD = Atherosclerotic Cardiovascular Disease, BAC = Breast artery calcification, CAC = coronary artery calcification. *Fisher's exact test

| Variable | OR (95% CI) |
| --- | --- |
| CAC (yes vs no) | 4.05 (1.36, 12.06) |
| BAC (yes vs no) | 4.27 (1.58-11.56) |
| BAC >10 (y vs n) | 13.04 (2.84-59.92) |
| CAC (continuous) | 1.19 (1.0, 1.44) |
| BAC (continuous) | 1.06 (1.01, 1.15) |

**Supplemental Table 2**: Odds Ratios of moderate-high ASCVD risk score (n=99). ASCVD = Atherosclerotic Cardiovascular Disease, BAC = Breast artery calcification, CAC = coronary artery calcification, CI = Confidence Interval, OR = Odds Ratio.
